# Managing the Earth’s Biggest Mass Gathering Event and WASH Conditions: Maha Kumbh Mela (India)

**DOI:** 10.1371/currents.dis.e8b3053f40e774e7e3fdbe1bb50a130d

**Published:** 2015-04-13

**Authors:** Annu Baranwal, Ankit Anand, Ravikant Singh, Mridul Deka, Abhishek Paul, Sunny Borgohain, Nobhojit Roy

**Affiliations:** School of Habitat Studies, Tata Institute of Social Sciences, Mumbai, Maharashtra, India; School of Habitat Studies, Tata Institute of Social Sciences, Mumbai, Maharashtra, India; Doctors For You, Mumbai, Maharashtra, India; Doctors For You, Guwahati, Assam, India; Doctors For You, Kolkata, West Bengal, India; Doctors For You, Guwahati, Assam, India; School of Habitat Studies, Tata Institute of Social Sciences, Mumbai, Maharashtra, India

## Abstract

Background: Mass gatherings including a large number of people makes the planning and management of the event a difficult task. Kumbh Mela is one such, internationally famous religious mass gathering. It creates the substantial challenge of creating a temporary city in which millions of people can stay for a defined period of time. The arrangements need to allow this very large number of people to reside with proper human waste disposal, medical services, adequate supplies of food and clean water, transportation etc. Methods: We report a case study of Maha Kumbh, 2013 which focuses on the management and planning that went into the preparation of Kumbh Mela and understanding its water, sanitation and hygiene conditions. It was an observational cross-sectional study, the field work was done for 13 days, from 21 January to 2 February 2013. Results: Our findings suggest that the Mela committee and all other agencies involved in Mela management proved to be successful in supervising the event and making it convenient, efficient and safe. Health care services and water sanitation and hygiene conditions were found to be satisfactory. BhuleBhatke Kendra (Center for helping people who got separated from their families) had the major task of finding missing people and helping them to meet their families. Some of the shortfalls identified were that drainage was a major problem and some fire incidents were reported. Therefore, improvement in drainage facilities and reduction in fire incidents are essential to making Mela cleaner and safer. The number of persons per toilet was high and there were no separate toilets for males and females. Special facilities and separate toilets for men and women will improve their stay in Mela. Conclusion: Inculcation of modern methods and technologies are likely to help in supporting crowd management and improving water, sanitation and hygiene conditions in the continuously expanding KumbhMela, in the coming years.

## Introduction

The World Health Organization (WHO) defines a mass gatheringas a “gathering of more than a specified number of persons at a definite location for a specific purpose for a defined period of time”. The number of persons may be as few as 1000, but the available literature suggests that gatherings exceeding 25,000 persons are considered to be a mass gathering.^1^ The large number of people attending a mass gathering makes planning and management of the event a substantial task. The organization of a mass gathering requires significant prior planning, to address the safety and needs of those who are staying in the event, prevent the spread of communicable diseases, and set up the management of health systems and other basic services. All this makes the process very complex.^2^

The name Kumbh Mela comes from Hindi, and originally from Sanskrit. *Kumbha *means *a pitcher* and *Mela *means *fair *in Sanskrit. The KumbhMela is the biggest and the most important periodic mass gathering in India. It is a mass Hindu pilgrimage of faith in which Hindus from all over the world gather to have a dip in a sacred river^3^ Kumbh Mela is held in four places every twelfth year by rotation- Haridwar in Uttarakhand (the Ganges), Allahabad in Uttar Pradesh (the confluence of the Ganges, the Yamuna and the mythical Saraswati), Nasik in Maharashtra (the Godavari) and Ujjain in Madhya Pradesh (the Shipra).^4^ The event requires the management of housing, transport, feeding and sanitation, security, crowd management, crime management, and trained manpower who can serve the large group of people.^5^ KumbhMela can be compared with the pilgrimage of Hajj in Mecca, which is another huge annual gathering and attracts millions of devotees to Mecca and its surrounding area. The management of Hajj is also a challenge, mainly because it involves large numbers of people who need to move in and around Mecca. There are various problems involved in the management of events such as Hajj due to the movement and residence of such large crowds.^6^ During KumbhMela, the government has to create a temporary city in which millions of people can stay for a defined period of time; without stampedes, epidemics, crimes and violence. The arrangements need to ensure that millions of people can reside with proper human waste disposal, medical services, adequate access to food, clean water and transportation. The Mela site is 80% submerged during the monsoon months and is only available for construction work during the two and a half months before the beginning of the KumbhMela.^7^ There are many good practices and gaps in the methods adopted by the management authorities for organizing mega events like Kumbh and Hajj.

We did a case study of MahaKumbhMela, 2013 which aims to examine the details of the management and planning of the event and to understand the water, sanitation and hygiene conditions of KumbhMela. We outline the overall management, which will help others to derive useful methods that can be adopted and replicated by the other states and the countries when organizing similar mass events. It might help to identify innovative methods to provide better water, sanitation and hygiene conditions to a mass gathering. The study provides a framework for similar research on future mass gathering events in India.

## Data and Methodology

In order to have complete understanding of the planning, management and understanding of water, sanitation and hygiene situations in KumbhMela 2013, field work was done for 13 days, from 21 January to 2 February 2013. Permission for conducting this study was obtained beforehand from all the concerned authorities and government officials who were involved in the Mela organization and management.

These steps were followed during the field work:

- **Interaction with the concerned authorities for understanding overall scenario of Kumbh:** Interaction took place with different doctors, municipal authorities, Disaster Relief force, fire service personnel to understand the overall scenario of Mela and to do social and resource mapping. The interaction was open ended with a focus on the measures which were applied by these authorities for organizing and managing the KumbhMela.
- **Transect walk (Observation), Social and Resource Mapping:** In this, the resources available for managing Mela were visited and observed. These included services such as health care centers, fire station, and water and sewage facilities. It was used to verify the claims of the different authorities providing services during the Mela. It used observation to map out the important available resources in the KumbhMela.
- **Water, Sanitation and Hygiene (WASH) survey:** To find out the status on water sanitation and hygiene conditions, a study was done in the Akharas/Santhas of KumbhMela. Akhara/Santhas is a Sanskrit word which denotes a place of practice with facilities for boarding, lodging,education and sports for a particular group of people. Akharas/Santhas were the places where the devotees temporarily stayed during the Mela. The water, sanitation and hygiene conditions of these Akharas are particularly important. In total, five Akharas/Santhas were randomly selected from each of the sectors. There were 11 sectors included in the study, which resulted in the sample size of 55 Akharas/Santhas. A close-ended questionnaire was used to interview each of the Akharas/Santhas. The questionnaire consisted of small set of questions, such as source of drinking water, water storage practices, defecation and hygiene practices in the Akharas/Santhas. The questionnaire was completed either by interacting with sadhus and people living there during the study or by observation of the researcher. The information regarding number of person and toilets in each of the Akharas/Santhas was obtained from those who were in charge of these Akharas/Santhas. Data analyses were done using Microsoft Excel and SPSS (version 20).

## Results

**Health Care interventions**

During the interaction with the Zonal Officer Health Department (ZOHD), we were informed that health care services became operational in mid November 2012, when approximately 250 doctors, including specialists and AYUSH (Department of Ayurveda, Yoga and Naturopathy, Unani, Siddha and Homoeopathy) doctors were deputed in the Mela area. Ten Sector hospitals had been constructed in the Mela area, each of which comprised of a general outpatient department (OPD) and 20 bedded inpatient unit. The hospitals were open for 24 hours every day throughout the duration of the Mela. Four MBBS (Bachelor of Medicine and Bachelor of Surgery) doctors were deputed in each sector hospital, with two doctors working in 8 hour shifts and two working in 12 hour shifts. In addition, nursing staff and one ambulance were also deputed for each sector hospital. The doctors arrived from the new Primary Health Centers in the other area of Allahabad and were assigned to Mela for two months. Each sector hospital had a pharmacy and medicines, which were provided free of charge. The daily OPD load was approximately 250-300 patients in each sector hospital. There were also 22 First Aid centers that had been set up by the health department in 12 sectors. Each first aid center had one AYUSH doctor, one pharmacist and a 2-bed inpatient unit. There were two infectious disease hospitals in the Mela area, with 20 beds in each. The focus of this healthcare delivery system was the central hospital in sector 2, where patients could be seen by a range of specialists, including Orthopedics, Medicine, Surgery, Eye, Skin and Obstetrics. There was a 100-bed inpatient unit and a 2-bed Intensive Care Unit. Diagnostic facilities such as X-ray, ultrasound, ECG (ElectroCardioGram) and a laboratory were also available. The additional directorate for health that supervised the entire healthcare delivery system was at the Mela field office, in Sector No. 2. More than70 ambulances and some river ambulances were deployed for transferring patients who needed specialized care from the First Aid Centers or Sector Hospitals to Central Hospital or Swaroop Rani Medical College. Safe passages had been constructed in the Mela area for the running of emergency vehicles during any emergency periods.

It was also noted that, in case of any mass casualty incident, 100 beds were reserved for the Mela in Swaroop Rani Medical College. No major health outbreaks occurred in the Mela area but the health department is ready to tackle any such outbreak by having a proper surveillance system in place. Illnesses such as fever, cold and cough were the most reported from the sector hospitals.

In addition to above facilities, the main focus areas of the health department were hospitals to provide health care to prevent vectors migrating from Allahabad city to the Mela area, the use of DDT (DichloroDiphenylTrichloroethane) spraying, fogging etc. to prevent vector breeding in the Mela area, and the provision and maintenance of sanitation facilities to all the Akharas/ Santhas and general public in the Mela area. The health department also looked after water quality in all their 22 circles, by conducting random orthotolidine tests twice every day.

**Water and Sewage interventions**

Unsafe water is the main cause of many water-borne diseases in mass gatherings like MahaKumbhMela. To prevent these diseases, water was another major focus area where the Government of Uttar Pradesh provided input. We were informed by those in-charge of the Uttar Pradesh Jal (water) Board, Sector 4, that the Jal board provides a running water supply for 24 hours every day, with a 550 km network of pipelines connecting all the Akharas/Santhas, hospitals, police and fire stations. A total of 42 high pressure pumps had been set up for this purpose. An average of 45 liters of water was provided per person per day. With the mission to provide safe and clean drinking water for the devotees, water quality was tested by Jal board, through an orthotolidine test of the supply water from 6 random sites. The Tata group of companies also worked with the Uttar Pradesh Jal Board to provide safe and clean drinking water to the devotees in KumbhMela. The Uttar Pradesh Jal Board was also responsible for sewage disposal in the Mela area. The board had constructed retention pools for sewage collection in the Mela area, so that sewage did not pollute the Sangam area.****

**Bhule Bhatke Kendra (Center for helping people who got separated from their families)**

The Kendra (center) is meant for those who get separated from their families during the mass gathering. At the KumbhMela, the kendra is marked by balloons with the words “BhuleBhatke Kendra”. Those in-charges of BhuleBhatke Kendra, Sector No. 4, informed us that there were 6 BhuleBhatke Kendra in the Mela area, as well as one camp where anybody who got lost was looked after. The devotees who got separated from their family members could come to kendra and report the incident. The Kendra then made a public announcement and displayed the picture of the missing person on their display panels. All information regarding the missing person was uploaded on their website which could be accessed by anyone. We were informed that 1560 missing persons were successfully located.****

**Fire stations**

****Fire incidents were frequent in the KumbhMela because of the cottages that were constructed with plastic sheets, inadequate temporary shelters, the use of LPG (Liquid Petroleum Gas) cylinders, and unplanned electrical lines near dwelling areas. The Fire Station Officer in the Mela area informed us that the department had set up 36 fire stations in the Mela area. They had 46 fire tenders with 422 firemen deployed in the Mela area. He added that the department has motorcycle mounted firemen with back pack fire extinguishers to provide emergency services to areas that could not be accessed by the fire tenders. The department also had an ambulance that could be used to transfer people from the incident site to the hospitals. They provided information on 18 major incidents of fire reported in the Mela. (See table 1)

**Table 1. Incidence of fire during the Mela by causes and number of person injured/ Died*:** *As reported by Fire stations****

Incident report: Serial no.	Cause	No. injured/Died
1.	Electric sparks	0
2.	Transformer Sparks	0
3.	Electric sparks	0
4.	Electric sparks	0
5.	Tent fire	0
6.	LPG leak	0
7.	Tent fire	0
8.	Halogen fire	0
9.	Short circuit	0
10.	LPG fire	0
11.	TransformerSparks	0
12.	Electric sparks	0
13.	LPG fire	25 Injured
14.	LPG fire	0
15.	Short circuit	0
16.	LPG fire	0
17.	Short Circuit	1 Died
18.	LPG Cylinder	2 Injured

**Disaster Response Team**

****To mitigate the risk of any kind of major incident, the Government of Uttar Pradesh deployed a National Disaster Response Team in the KumbhMela. The National Disaster Relief Force team had been deployed to aid in search and rescue operations, if necessary. The team had specialized diving equipment and experienced personnel for such operations and was available throughout the course of the KumbhMela. The battalion comprised of navy and paramilitary forces, who had been given one month’s special training before being sent to NDRF (National Disaster Response Force). Other paramilitary forces were also deployed in the Mela area.****

**WASH interventions**

****Water and sanitation facilities were maintained by the Mela Committee. All the Akharas was provided with toilets depending upon the number of people staying in each Akharas. We observed that every Santha/Akhara was provided toilets from Mela committee and sweepers were also allotted for each Santha/Akhara.

**WASH Survey**

****In all the Akharas, water for drinking and other uses was the water supply provided by the Uttar Pradesh Jal board (table2). Buckets were used to store the water in these Akharas and all water containers were also clean. Similarly, all the Akharas had latrines which were used by both males and females. All Akharas had a garbage pit for waste disposal. Our findings were consistent with earlier observation and claims of the Uttar Pradesh Jal Board. However, drainage was a major problem in 45% of Akharas. We found that 42% of Akhara/Sanstha had 11-15 persons per toilet and 30% of Akharas/Sansthas had more than 15 persons per toilet (figure 1).

**Percentage distribution of Akshara/Santhas by person per toilet:**
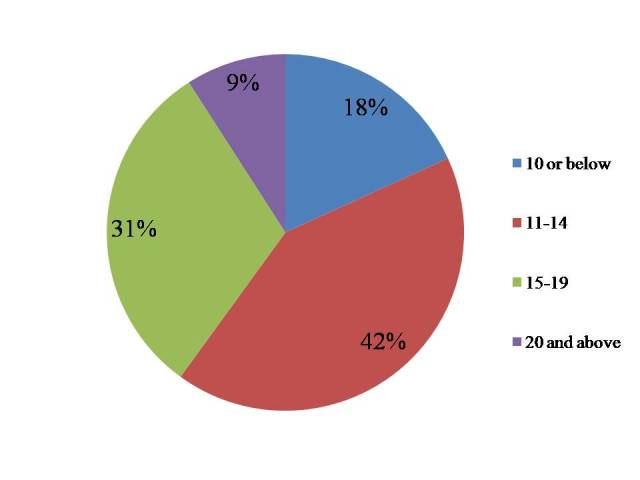

**Table 2. Percentage of Akhara/Sanstha (N=55) by WASH conditions:** 

Source of water for drinking, domestic use and personal hygiene was the provided water supply	100
Buckets were used for storing water	100
Bucket or container has a lid	72.7
Water container was cleaned	100
Had latrine	100
Had a designated area for defecation	27.3
Latrine was used by both males and females	100
Had garbage pit for waste disposal	100
Vector control was not a major problem	100
Had a drainage problem	45.5

## Discussion

Our study shows that there was high quality planning and administration in the KumbhMela 2013. Mela committee and all other agencies involved in Mela management were successful in the supervision of the event and in making it convenient, efficient and safe. Health care services and water sanitation and hygiene conditions were found to be satisfactory. Our findings are consistent with the claims made by the Uttar Pradesh Jal board and previous studies done on KumbhMela. One of these studies stated that after seeing the top quality arrangements of KumbhMela, the world witnessed the administration of Mela with awe and surprise; and considered the planning and management of KumbhMela as one of the wonders of modern day management.^7^ Another study supports Mela administration, police and other involved agencies for their efficient planning and management, including cost and service management to make MahaKumbh a mishap free event.^8^ In many ways, the 1,200-hectare township of Mela was not dirty. It did not have garbage disposed of everywhere, and the low number of mosquitoes and flies made it safer to live. Around 6,000 sweepers worked round the clock to clean the area. Around 200 tonnes of solid waste was removed from Kumbh every day. The authorities arranged for 80 million liters of drinking water, 40,000 portable toilets, 243 doctors and treated more than 100,000 people for various infections at the14 hospitals in the Mela area.^5^ As per the findings of the present study, BhuleBhatke Kendra completed the major task of finding missing people and helping them to reunite with their families.

Some shortfalls were that drainage was found to be a major problem and some fire incidents were reported, which needs serious attention from the Mela organizers. Improvement in drainage facilities and reduction in fire incidents are basic but essential tasks to make Mela cleaner and safer. A higher number of persons per toilet than would be preferred were found and there were no separate toilets for males and females. Special facilities and separate toilets for men and women would improve their stay in the Mela, and will help to improve the public health conditions and increase the effective use of toilet facilities provided. Inculcation of modern methods and technologies can help in supporting the crowd management and improving water, sanitation and hygiene conditions in the continuously expanding KumbhMela, in the coming years. For example, Kumbh could be improved with strategies from Hajj. In the past few years in Hajj, different kinds of sensor devices, scanners, trackers of footsteps, modern medical and emergency related services and many other innovative technologies were found to improve its management.^6^

## Conclusions

Despite the shortfalls, one must appreciate that the government has done a great work in managing this unique event. Additionally, it has adopted some of the best practices that have captured the attention of many researchers, governments, and international research firms, not only in India but around the world. The management of KumbhMela provides an example to follow for mass gathering events inside and outside India, showing how a mass event can be well organized with utmost safety security and cleanliness. Further research is needed on KumbhMela to bring into focus the strengths and weaknesses of this large event for its further improvement.

## Competing Interest

The authors have declared that no competing interests exist.
